# Revealing Topological Barriers against Knot Untying in Thermal and Mechanical Protein Unfolding by Molecular Dynamics Simulations

**DOI:** 10.3390/biom11111688

**Published:** 2021-11-13

**Authors:** Yan Xu, Runshan Kang, Luyao Ren, Lin Yang, Tongtao Yue

**Affiliations:** 1 College of Electronic Engineering and Automation, Shandong University of Science and Technology, Qingdao 266590, China; xuyan2020@sdust.edu.cn; 2 College of Chemical Engineering, China University of Petroleum (East China), Qingdao 266580, China; s20030096@s.upc.edu.cn; 3 Key Laboratory of Marine Environment and Ecology, Institute of Coastal Environmental Pollution Control, Ministry of Education, College of Environmental Science and Engineering, Ocean University of China, Qingdao 266100, China; aaarlyao@163.com (L.R.); yanglin9005@stu.ouc.edu.cn (L.Y.); 4 Laboratory for Marine Ecology and Environmental Science, Qingdao National Laboratory for Marine Science and Technology, Qingdao 266237, China

**Keywords:** knotted protein, folding/unfolding, knot untying, molecular dynamics simulation

## Abstract

The knot is one of the most remarkable topological features identified in an increasing number of proteins with important functions. However, little is known about how the knot is formed during protein folding, and untied or maintained in protein unfolding. By means of all-atom molecular dynamics simulation, here we employ methyltransferase YbeA as the knotted protein model to analyze changes of the knotted conformation coupled with protein unfolding under thermal and mechanical denaturing conditions. Our results show that the trefoil knot in YbeA is occasionally untied via knot loosening rather than sliding under enhanced thermal fluctuations. Through correlating protein unfolding with changes in the knot position and size, several aspects of barriers that jointly suppress knot untying are revealed. In particular, protein unfolding is always prior to knot untying and starts preferentially from separation of two α-helices (α1 and α5), which protect the hydrophobic core consisting of β-sheets (β1–β4) from exposure to water. These β-sheets form a loop through which α5 is threaded to form the knot. Hydrophobic and hydrogen bonding interactions inside the core stabilize the loop against loosening. In addition, residues at N-terminal of α5 define a rigid turning to impede α5 from sliding out of the loop. Site mutations are designed to specifically eliminate these barriers, and easier knot untying is achieved under the same denaturing conditions. These results provide new molecular level insights into the folding/unfolding of knotted proteins.

## 1. Introduction

Understanding how proteins fold into their native states is of essential importance for tackling diseases via elucidation of pathology caused by protein unfolding or misfolding [1]. We have learned from decades of experimental and computational studies that both protein folding and functioning are essentially determined by the unique information encoded in their amino acid sequences [2,3,4]. Based on this theory, the artificial intelligence model of AlphaFold was developed, which can accurately predict static structures of most proteins given only their amino acid sequences [5,6]. By contrast, the dynamic information about how proteins fold into their native states is relatively less understood [7]. In particular, our past knowledge on protein folding has been challenged since identification of knotted topologies, which play essential roles in protein functioning, such as providing additional stability for maintaining the global fold and catalytic properties [8,9,10,11,12,13]. Elucidation of the folding mechanisms of knotted proteins represents an important new challenge in the protein-folding field.

Knotted proteins need to overcome topological barriers to reach their functional native states, and their folding should be slow [14,15]. Slipknotting, threading and mouse trapping are several possible pathways revealed for knotting of proteins during folding [16,17,18]. By means of ensemble measurements, Jackson et al. demonstrated that knotted proteins can spontaneously fold into their native knotted structures, and knotting is the rate-limiting step that occurs early in the folding reactions [15]. The view of knotting prior to folding was indirectly supported by a finding that the methyltransferases (MTases) remained knotted in their chemically denatured states, and the denatured protein can efficiently refold [19,20]. Of note, it is quite difficult to achieve untying of knots during in vitro denaturation studies, because the kinetics of knot untying is generally orders of magnitude slower than protein unfolding [21]. This indicates that, with extended time under denaturing conditions, it is possible for proteins to be unfolded with their knots untied, possibly in a stepwise process [21,22]. However, information about how knots are spontaneously formed during protein folding, or untied in protein unfolding, remains largely unknown.

In comparison to ensemble methods, single-molecule force spectroscopy (SMFS) is advantageous in manipulating the knot/slipknot structures by stretching proteins on specific points with controlled forces [23,24,25,26]. In particular, atomic force microscopy (AFM) was mainly used to study the mechanisms underlying mechanical unfolding and untying of knotted/slipknotted proteins, but the relatively low force resolution (~10 pN) restrains its usage for direct observation of protein folding [24,25,27]. By contrast, the optical tweezer (OT)-based SMFS has a higher resolution in force (~0.1 pN) and has been utilized to investigate folding/unfolding mechanisms of some knotted proteins [23,26,28]. By means of AFM and OT based SMFS, Li et al. analyzed the force–distance curves in correlation with structural changes during mechanical tying, untying and tightening of knots in proteins, and revealed two- and three-state pathways of folding and unfolding of knotted/slipknotted proteins [26,29]. These experimental studies provided mechanistic insights into the folding of knotted proteins. Nevertheless, due to complexity and variability of the intramolecular interactions, revealing barriers against knot untying in protein unfolding continues to be a substantial challenge, requiring advanced analytical techniques with high-resolution and high-precision in both spatial and temporal scales.

Molecular dynamics (MD) simulation serves as an important complementary method of experiments to reach a deeper understanding of the molecular mechanisms of protein folding and unfolding [25,30]. Using unbiased all-atom MD simulations, Noel et al. demonstrated that proteins can spontaneously fold to reach the knotted native state initiated from unknotted or slipknotted intermediates [17]. Steered MD simulations suggested formation of a slipknot structure as an important intermediate step to reduce the topological difficulty as knotted proteins fold [25]. Combining single-molecule fluorescence resonance energy transfer and MD simulations, the knot in TrmD was found to slide towards the C-terminal during unfolding, and the knot size kept nearly unchanged [31]. Our previous simulations showed that the knot can restrict surrounding domains to retard opening of the hydrophobic core, which was defined as a crucial step of protein unfolding [11]. Moreover, the knotted conformation was found to cooperate with dimerization to further enhance the protein stability [12].

In this study, we chose the methyltransferase YbeA from *Escherichia coli* as the knotted protein model, combining unbiased and steered MD simulations to analyze thermal and mechanical protein unfolding and related changes in the knotted conformation. To answer whether and how the knot can be untied in protein unfolding, we firstly performed five independent simulations under the same enhanced thermal denaturing conditions totaling over 5 μs. YbeA in only one out of five simulations reached the denatured state with the knot completely untied through knot loosening, while in other simulations the knot was well maintained. Inspired by the nanopore designed for DNA and protein sequencing [32,33], we employed a single-walled carbon nanotube of a diameter smaller than the tightened knot size for steered protein translocation through the tube from the C- and N-terminals of the protein. Through analyzing force spectrum and changes of both the knot position and size in correlations with protein unfolding, four aspects of intramolecular forces against knot untying were revealed. We designed site mutations to specifically reduce these barriers, and achieved steady knot untying and sliding under the same denaturing conditions. Although YbeA has the relatively simple trefoil knot among family of knotted proteins, the revealed mechanisms can be applied to other proteins to help elucidate folding/unfolding of knotted proteins.

## 2. Materials and Methods

### 2.1. Models

**Figure 1:**
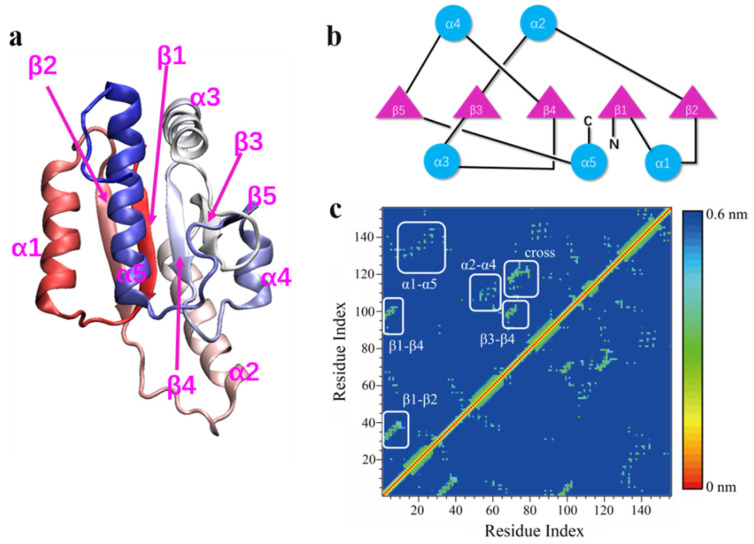
Structural analysis of the monomeric YbeA used in our simulations. (**a**) Equilibrium structure of the monomeric protein with a color gradient from N-terminal (red) to C-terminal (blue). (**b**) Topological diagram showing relative positions and connections of different domains and how the knot is formed. (**c**) Contact map of the monomeric YbeA, which was plotted according to the shortest distance between each pair of residues inside the protein.

*Escherichia coli* methyltransferase (MTase) YbeA is a 155-residue protein possessing a trefoil knot within its backbone structure (Figure 1a). The knot is formed by threading the last 35 residues (120–155, α5) through a loop formed by 49 residues (70–119, β3-α3-β4-α4-β5) (Figure 1b). YbeA belongs to the subclass of α/β-knotted MTases, a type of enzyme catalyzing transfer of the methyl groups of S-adenosyl methionine to carbon, nitrogen, or oxygen atoms in DNA, RNA, proteins, and other small molecules [34]. By contrast with other knotted proteins, YbeA has a relatively simple structure with a proper molecular size, making it an ideal model for investigations of folding/unfolding mechanisms of trefoil-knotted proteins [15,19].

### 2.2. Molecular Dynamics Simulation

Simulations presented in this work were conducted based on the all-atom molecular dynamics (MD) method, which has been widely used for studies of biological systems, including proteins, DNA and plasma membranes [35,36,37,38,39,40,41,42]. Equilibrium and thermal denaturation simulations were performed using the GROMACS software package (KTH Royal Institute of Technology, Stockholm, Sweden) version 2019.3 on GPU [43], which greatly improved computational efficiency. Mechanical denaturation simulations were performed using the GROMACS software package version 4.6.7, with explicit solvent [44]. The CHARMM27 force field was employed for proteins [45], together with the TIP3P model for water [46]. Although most MTases containing trefoil knots usually exist and function in nature as homodimers, previous studies have demonstrated the stability of the monomer [11,12,16]. Besides, it was proved that trefoil knotted proteins usually fold into their native states in the monomer before dimerization in order to function [47]. Therefore, we chose the monomeric YbeA in our simulations for the study of knot untying in protein unfolding. The all-atom protein structure of YbeA was separated from the dimer taken from the Protein Data Bank (PDB ID code 1NS5). Based on the native structure, we designed site mutants (Trp120, Pro128 and Pro130 replaced with Ala) for unbiased and steered simulations to manifest the barriers against knot untying in protein unfolding.

To prepare the simulation system, the knotted protein was positioned at the center of a cubic box of size 10 nm × 10 nm × 10 nm filled with 31894 water molecules and 122 ions (62 Cl^−^ and 60 Na^+^), representing the ionic concentration of approximately 0.1 M. To remove bad initial contacts that may impact subsequent equilibrium simulations, the system energy was first minimized using the steepest descent method. Then, we performed a short simulation of 10 ns with the canonical ensemble (constant atom number, box size and temperature), during which the heavy atoms in the protein were restrained to move, thus allowing solvent molecules to fully dissolve the protein. After that, both coordinates and velocities acquired in the previous step were used as the starting point for subsequent 10 ns simulations with the NPT ensemble, during which the heavy atoms in protein were also restrained. Pressure was kept constant at P = 1 bar using the Parrinello–Rahman barostat [48], and temperature was kept at T = 300 K using the v-rescale thermostat [49], with a coupling constant τ = 2 ps. After completion of the two phases, production MD run of 1000 ns with NPT ensemble for data collection was performed without position restraint. Non-bonded interactions were truncated at a cutoff of 1.0 nm, and the particle-mesh Ewald summation method was used to treat the long-range electrostatic interactions [50]. All simulations were performed with a time step of 2 fs, and the neighbor list was updated every 10 steps. Periodic boundary conditions were considered in all three directions. Snapshots were rendered using VMD (version 1.9.2, University of Illinois at Urbana-Champaign, Urbana, IL, USA) [51].

### 2.3. Thermal Denaturation

To accomplish thermal denaturation of the knotted protein by simulations, the system temperature was set to 520 K. Although the temperature set in simulations was higher than that used in experiments, it was chosen so as to accelerate the unfolding to be affordable by all-atom MD simulations [52]. This strategy was applied by other researchers, demonstrating that simulations under harsh conditions provide results applicable to normal denaturing conditions [53,54]. The critical temperature of the TIP3P water for phase transition is 578 K, and vapor and liquid phases may coexist at 520 K according to the vapor–liquid equilibria [55]. Although using the NVT ensemble would prevent box expansion under higher temperatures, the system pressure would strikingly increase to generate artifacts. Thus, all thermal denaturation simulations presented in this study were conducted with the NPT ensemble.

### 2.4. Mechanical Denaturation

To perform steered MD simulations, the box size was increased to 10 × 10 × 60 nm^3^ to allow exertion of an external force on one terminal of the protein along the z direction, and avoid self-interactions of the unfolded protein with its periodic neighbors. The single-walled carbon nanotube (SWCNT) with a diameter of 1.0 nm and length of 2.0 nm was constructed via the nanotube builder plugin of the VMD software package (version 1.9.2, University of Illinois at Urbana-Champaign, Urbana, IL, USA) [51], with the force field parameters acquired from previous studies [56]. In particular, the equilibrium carbon–carbon bond lengths were 0.1375 nm, maintained by harmonic potentials with spring constants of 30,500 kcal mol^−1^ nm^−2^. The equilibrium bond angles were 120°, maintained by harmonic potentials with spring constants of 40 kJ mol^−1^ rad^−2^. Before simulations of protein translocation through SWCNT, processing simulations were performed to ensure that the defined terminal residues can enter SWCNT. Firstly, the Cα atom of either terminal residue was pulled with a constant velocity (v = 0.0005 nm/ps) along the direction of tube axis, while residues 9–155 or 1–148 were restraint in their coordinates in this period. When the residues without restraint (1–8 or 149–155) were totally outstretched, SWCNT was positioned along the defined direction with its entrance nearing the Cα atom of either terminal residue of the protein. Then the Cα atom of either terminal residue was pulled with a constant velocity (v = 0.0005 nm/ps) along the tube axis. All atoms in the SWCNT were fixed at their initial positions. This mechanical manipulation has been widely applied to investigate protein unfolding and DNA/protein sequencing [32,33,57,58,59,60].

### 2.5. Knot Detection Algorithm

Tracking changes in knot position and size is of central importance to elucidate how the knot is untied or maintained during thermal and mechanical protein unfolding. In our simulations, the trajectory file was uploaded to the KnotProt 2.0 database to analyze the knot change [61,62,63]. In this database, several steps are required to detect knot and analyze the knot type, size and position. Firstly, to detect a knot type, an open chain was transformed to a closed chain by applying a random closure method. Then, the KMT algorithm was applied to reduce the closed terminals to a shorter configuration [64]. This algorithm analyzes all triangles in a chain made of three consecutive amino acids, and removes the middle amino acid in case a given triangle is not intersected by any other segment of the chain. After a number of iterations, the initial chain is replaced by a much shorter chain of the same topological type. Then the knot types resulting from individual closures are determined by computing Alexander polynomial knot invariants.

## 3. Results

### 3.1. Knot Untying under Enhanced Thermal Fluctuations

Although most α/β-knotted proteins, including YbeA considered in our simulations, exist and function in nature as homodimers, previous studies have demonstrated stability of the monomeric protein owing to the inherent knot [10,11,17]. Dimerization provides more stability via cooperating with the knotted conformation, as revealed by our previous simulations [12]. In this study, the monomeric protein was employed to analyze knot conformational change under denaturing conditions. Firstly, one unbiased simulation was performed under normal conditions (0.1 M NaCl, T = 300 K). Consistent with our previous study [11], the monomeric protein was quite stable in the finite simulation period (600 ns), as evidenced by time evolutions of the native contact ratio, root-mean-square deviation (RMSD), and secondary structure changes (Figure S1). The shortest distances between pairs of residues in YbeA were calculated and summarized in the contact map (Figure 1c). In accordance with the equilibrium structure and the topological diagram (Figure 1a,b), six major contact regions were identified inside the protein, including α1–α5, α2–α4, β1–β4, β1–β2, β3–β4, and the knot crossing region.

After reaching equilibrium, the system temperature was increased to 520 K to examine changes in the knot position and size under enhanced thermal fluctuations. Although the temperature set in simulations was higher than that used in experiments, it was chosen so as to accelerate the unfolding to be affordable by all-atom MD simulations. This strategy has been implicated in previous studies [65]. To perform thermal denaturation simulations at realistic temperature, the latest version of GROMACS can be used especially in the use of GPU graphics cards. Alternatively, it can perform the simulations in a coarse-grained model. Considering the stochastic nature of protein unfolding [66], five independent simulations under the same denaturing condition were performed, each lasting over 1.0 μs. In all simulations, the protein’s native contact ratio decreased from 1.0 to about 0.1 (Figure S2a), and the RMSD increased from 0 to above 1.5 (Figure S2b). Ratios of secondary structures (mostly α-helices and β-sheets) were calculated, suggesting that most β-sheets were denatured, and α-helices kept fluctuating (Figure S3a–e). That was distinct from the case under normal conditions (Figure S3f), suggesting that the knotted protein YbeA had been denatured under enhanced thermal fluctuations.

**Figure 2:**
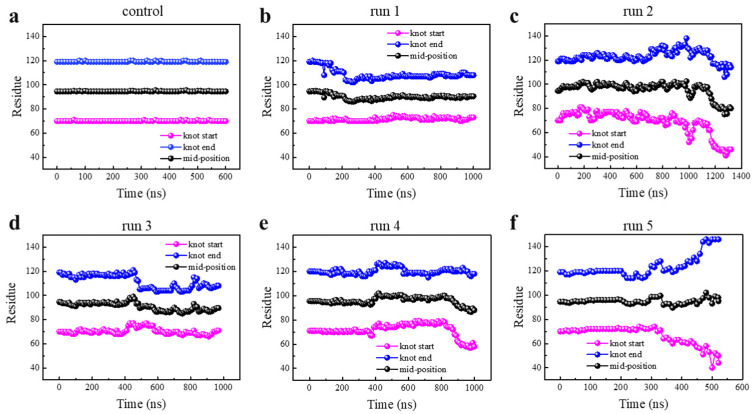
Sequential movement of two knot termini and the corresponding mid-position for YbeA under the normal (T = 300 K, (**a**)) and thermal denaturing (T = 520 K, (**b**–**f**)) conditions. Five independent simulation runs were performed under the same enhanced thermal denaturing conditions.

Although protein was denatured under enhanced thermal fluctuations as manifested, the above data cannot reflect whether the knot was untied or maintained during the protein unfolding process. The trajectory file was uploaded to the KnotProt 2.0 database to monitor changes in the knot position and size during each protein unfolding process (Figure 2, details are given in Models and Methodology). As is seen, the knot kept nearly unchanged under normal conditions (Figure 2a), while under enhanced thermal denaturing conditions, all knots fluctuated in their position and size (Figure 2b–f). Of note, the knot was untied in one out of five simulations (run 5, Figure 2f), but kept tied during and after denaturation in other simulations (runs 1–4, Figure 2b–e). In these four simulations, the knot mid-position was found to move slightly toward the N-terminal. The knot size was determined through identifying two knot termini, and found to be slightly tightened in simulation runs 1 and 3, but slightly loosened in runs 2 and 4 (Figure S4). Especially in run 2, the knot loop expanded towards both terminals at 1000 ns; it seems to be a high possibility that the knot would be untied in an extended simulation, but the results did not reach our expectation in 400 ns. In run 5, two ends of the knot slid toward opposite directions (Figure 2f), suggesting that the knot was untied through knot loosening rather than sliding toward either direction (the continuous process of knot untying during protein unfolding can be found in Video S1), consistent with previous experiments by Wang et al. using single-molecule force spectroscopy [29].

**Figure 3:**
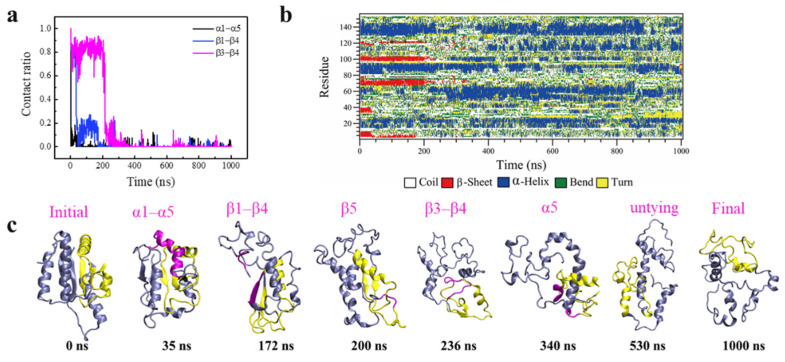
Simulation run 5 of the thermal protein unfolding, during which the knot was untied. (**a**) Time evolutions of the ratio of native contacts between selected domains of α1 and α5, β1 and β4, β3 and β4. (**b**) Time evolutions of the YbeA’s secondary structure change. (**c**) Time sequence of typical snapshots depicting the stepwise change of the protein structure under enhanced thermal fluctuations. The knotted region is colored in yellow. The major secondary structural change at each step is colored in purple.

The knot untying, despite of low probability in our simulations, was unexpected because the kinetics of knot untying was estimated at least an order of magnitude slower than protein unfolding [21]. As demonstrated in our previous studies, separation between domains α1 and α5 was a crucial leading step of protein unfolding via opening the hydrophobic core composed of β-sheets [11,12]. Considering that the knot is formed by threading α5 through a loop formed by β3-α3-β4-α4-β5 (Figure 1b), the ratio of native contact between two selected domains was calculated to monitor the detailed structural changes of the protein (Figure 3a). Combining time evolutions of the knot position and size (Figure 2f) and the secondary structure changes (Figure 3b), the stepwise process of protein unfolding followed by knot untying can be manifested. Clearly, the knot was beginning to untie from ~300 ns (Figure 2f), but most protein’s secondary and tertiary structures had been denatured before this time point. In particular, opening of the hydrophobic core via separation between α1 and α5 was first observed at t = 35 ns (Figure 3c). As α1 was in direct connection with β1, which formed favorable contacts with β4 (Figure 1b), the loss of α1 contact with α5 relieved its constraint on β1, thus reducing contact ratio between β1and β4 to 0 at t = 170 ns (Figure 3a). As a consequence, the knotted region was separated from the unknotted region at t = 172 ns (Figure 3c). Subsequently, β5 was denatured at t = 200 ns (Figure 3b), and contacts between β4 and β3, which constituted the loop through which α5 was threaded to form the knot, were perturbed at t = 236 ns (Figure 3a). After denaturation of these β-sheets that caused loop loosening, α5 was denatured at t = 340 ns (Figure 3b), and the knot was untied in a short simulation time.

### 3.2. Knot Maintenance in Thermal Protein Unfolding

Although the knot in YbeA was occasionally untied under enhanced thermal fluctuations as described above, the knot was mostly maintained in four out of five independent simulations. It suggests barriers against knot untying in protein unfolding. The protein’s structural changes in other parallel simulation runs were analyzed to reveal these barriers and elucidate how the knot kept tied in thermal protein unfolding. Taking run 1 as an example, we also observed separation between α1 and α5 as the first step to open the hydrophobic core composed of β-sheet domains (Figure S5). Similar to run 5, in which the knot was untied, the α1-α5 separation was followed by separation between β1 and β4 at t = 130 ns. Notably, once β5 was denatured at t = 140 ns, the end point of the knot started to move toward the N-terminal with the start point nearly unchanged to decrease the knot size (Figure 2b). Different from the simulation run 5 in which the contacts between β3 and β4 were broken early to enlarge the knot loop, these two domains kept in touch to resist their unfolding (Figure S5). Thus, the loop was not enlarged with the knot maintained in the finite simulation time. In other simulation runs, the protein’s structural change followed the similar sequence, differing in the time points for these cascading events (Figures S6–S8). Overall, the knot could be finally untied only if all constraints maintaining the topological knot were destroyed at certain points under enhanced thermal denaturing conditions. Otherwise, the knot would keep tied in sufficient time, consistent with previous experimental results [19,20].

The trefoil knot of YbeA is located in a shallow position, near its C-terminal in the protein sequence, and is formed by threading α5 through a loop formed by β3, β4 and α3 (Figure 1b). We thus expected knot untying through sliding toward the C-terminal which, however, did not occur in all simulation runs. Apart from the restrained loop size by favorable interactions inside the hydrophobic core as elucidated above, there should exist other forces that resist sliding of α5 out of the loop under thermal denaturing conditions. Through analyzing the amino acid sequence coupled with the local secondary structure, two proline residues (Pro128 and Pro130) were identified at the N-terminal of α5. When proline is in a peptide bond, it cannot donate a hydrogen atom to form a hydrogen bond that stabilizes an α-helix or a β-sheet. Since the three-carbon R-group of proline is fused to the α-nitrogen group, this compound has a rotationally constrained rigid-ring structure. Thus, proline has the strongest turn-forming propensity of all amino acids [67,68]. We calculated the dihedral formed by Cα atoms of Pro128, Pro130 and Arg133, and found a narrow distribution of the angle between 90°and 120°, with an average value of 108° (Figure S9). It suggests that α5 has a relatively rigid turning at its N-terminal that cooperated with interactions with α1 to hinder its sliding out of the loop.

### 3.3. Steered Translocation of YbeA through SWCNT

Steered MD simulations have been previously conducted in combination with AFM and OT-based SMFS to reveal kinetics of mechanical protein unfolding and knot tightening [25,27,28]. To further manifest and elucidate the barriers against knot untying, we got inspiration from DNA/protein sequencing through a nanopore, and designed protein translocation through a SWCNT. As reported by earlier experimental and simulation studies, the radius of gyration of the tightened knot, R_g_, is about 0.72 nm [59]. We thus set the SWCNT diameter as 1.0 nm, which is smaller than 2R_g_, to prevent translocation of the tightened knot across the pore. As such, we expected two outcomes of steered protein translocation through the SWCNT, i.e., the knot would either slide toward the opposite direction of pulling or be tightened and stuck at the opening of the SWCNT. Through analyzing and comparing the mechanical responses of the same protein under two different pulling directions, the barriers against knot untying can be manifested.

**Figure 4:**
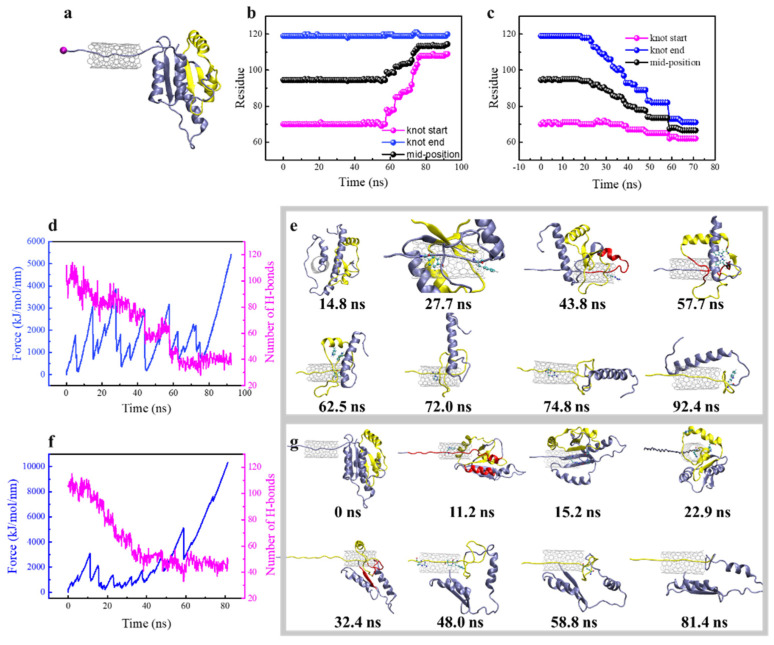
Steered MD simulation of YbeA translocation through SWCNT. (**a**) The simulation system setup illustrating the steered protein translocation through SWCNT. (**b**,**c**) Sequential movement of two knot termini and mid-position for YbeA when the N- (**b**) and C-terminal (**c**) were pulled through SWCNT. (**d**) Time evolutions of the pulling resistant force and hydrogen bond number when pulling the N-terminal through SWCNT. (**e**) Time sequence of typical snapshots depicting the protein structural response to generate resistant forces identified in (**d**). (**f**) Time evolutions of the pulling resistant force and hydrogen bond number when pulling the C-terminal through SWCNT. (**g**) Time sequence of typical snapshots depicting the protein structural response to generate resistant forces identified in (**f**).

The steered protein translocation through SWCNT is illustrated in Figure 4a (Videos S2 and S3). Shown in Figure 4b,c are changes of the knot position in response to protein translocation through the SWCNT from the N-terminal and C-terminal, respectively. When the N-terminal (Cα atom of Met1), which is relatively distant from the knot, was pulled through the tube, the knot position kept nearly unchanged in the first 50 ns, and 65 residues were pulled into SWCNT in this period (Figure 4b). After that, the knot start-point close to the N-terminal moved toward the C-terminal, while the end-point kept unchanged. Thus, the knot was gradually tightened to 12 residues and slid towards the C-terminal in about 20 residues in the whole simulation period. By contrast, when the C-terminal closer to the knot was pulled, the knot responded earlier (t = 20 ns) and slid towards the N-terminal in about 9 residues. The knot was finally tightened into the same size. According to the protein’s secondary structure changes, the protein was sequentially unfolded as pulled into the pore, and the knot termini started to move toward the opposite direction of pulling upon reaching the SWCNT (Figure S10). The pulling resistance force, number of H-bonds, and corresponding protein’s structural changes were recorded to explain the difference (Figure 4d–g). As is seen, there are several force peaks during steered protein translocation through the tube, and the mechanical response was quite different between two pulling directions. Through analyzing structure changes for stretching the N-terminal, the force peaks respectively at t = 5 ns, 15 ns, 28 ns and 42 ns represent Leu3 entering the tube, α1-α5 separation, Tpr23 and Phe27 entering the tube, and unfolding of α2 helix (Figure 4e), which were accompanied by the sudden drops of the H-bond number (Figure 4d). Above force peaks correspond to mechanical unfolding of the unknotted region of the protein. After that, the knot started to change, and the force peak at t = 57 ns was generated by the destruction of H-bonds and hydrophobic interactions between β3, β4 and β5. Upon separation of these β-sheets composing the hydrophobic core, the π-π interactions between residues Trp79 and Trp120 stabilizing the knot crossing of C-terminus were destroyed, as reflected in a sudden increase of distance between Trp79 and Trp120 at t = 60 ns (Figure S11a). After that, the π-π interactions between Trp91 and Trp120 that stabilized the loop were perturbed to generate a force peak at t = 62.5 ns (Figure 4e). Then, the knot was tightened gradually after crossing a barrier due to the obstruction of residues Arg90 and Arg96. At the end, the tightened configuration of the trefoil knot restrained its entrance into the tube, thus generating a striking and continuous increase of the resistance force from t = 80 ns. Notably, it was the bulky aromatic side chain in Trp120 that hindered sliding of the tightened knot towards the C-terminal (Figure 4e, t = 92.4 ns).

Considering that the trefoil knot of YbeA is located nearer to its C-terminal, pulling of the Cα atom of the C-terminal (Cα atom of Glu155) was expected to generate different mechanical responses and structural changes of the same knotted protein (Figure 4f,g). The first dominant force peak at t = 11.2 ns was generated by mechanical unfolding of α5 during its separation with α1 and translocation through the tube. As the N-terminal of α5 reached the tube entrance, the rigid turning maintained by Pro128 and Pro130 as identified above was deformed for translocation, thus generating a force peak at t = 15.2 ns. After t = 18.2 ns, the Trp120-led β5 reached the opening of the SWCNT and was trapped there due to the aromatic group. To slide into the tube, Trp120 was forced to separate from Trp79 and Trp91 (Figure S11b), thus generating a force peak at t = 22.9 ns. Then, α4, β4 and α3 were unfolded sequentially, accompanied with separation between β4 and β1 as reflected by a sharp decrease of the H-bond number at t = 32.4 ns. This also represents separation between the knot region and the unknotted region. The force peak at t = 58.8 ns was induced by Gln83 trapped inside the loop at the entrance of the tube. In this period, the knot slid towards the N-terminal after overcoming several barriers caused by interactions between bulky residues and the tube surface. At last, the knot was tightened and got stuck at entrance of the tube. These results suggest that the sliding pathway for knot untying of YbeA could be eliminated.

### 3.4. Four Aspects of Barriers Jointly against Knot Untying in Protein Unfolding

**Figure 5:**
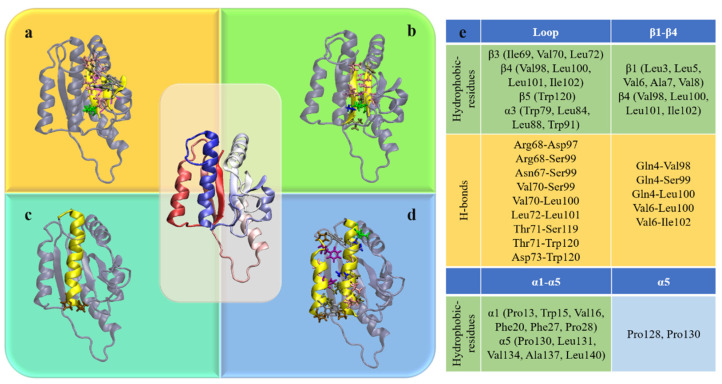
Four aspects of barriers against knot untying in protein unfolding. (**a**) Interactions between β4, β3 and β5 preventing the loop from loosening. (**b**) β1-β4 interactions. (**c**) Rigid turning of α5 defined by residues Pro128 and Pro130. (**d**) α1-α5 interactions protecting the hydrophobic core. (**e**) Summary of residue interactions contributing to these barriers.

The dynamic processes of protein folding/unfolding coupled with knot tying/untying involve complex intramolecular interactions with accompanied topological changes that are difficult to study using experimental techniques. Summarizing data acquired from above thermal and mechanical unfolding simulations, four aspects of intramolecular forces against knot untying in protein unfolding can be revealed, as illustrated in Figure 5. Firstly, in terms of the knot itself, interactions between β4, β3 and β5 stabilize the knot loop and prevent it from loosening. In particular, hydrophobic (e.g., Ile69, Val70 and Leu72 in β3, Val98, Leu100, Leu101 and Ile102 in β4, and Trp120 in β5) and hydrogen bonding (six H-bonds between β4 and β3, three H-bonds between β3 and β5) interactions are formed inside the loop. Besides, Trp79 in β3 and Trp91 in α3 form strong π-π interactions with Trp120 in β5 to further enhance the loop stability (Figure 5a). Secondly, β1 outside the knot region forms hydrophobic interactions (e.g., Leu3, Leu5, Val6, Ala7, Val8) and five hydrogen bonds with β4, providing stability of the start terminal of the knot (Figure 5b). Thirdly, the rigid turning of α5 defined by residues Pro128 and Pro130 prevents it from bending and sliding out of the loop (Figure 5c). Finally, hydrophobic interactions between α1 and α5 (Pro13, Trp15, Val16, Phe20, Phe27 and Pro28 in α1, Pro130, Leu131, Val134, Ala137 and Leu140 in α5) further enhance the knot stability through protecting the hydrophobic core composed of β-sheets (Figure 5d). Residues involved in these barriers against knot untying in protein unfolding are summarized in Figure 5e.

It is of note that the knot untying in YbeA unfolding cannot be restrained by any single barrier manifested above. We expect that different barriers may cooperatively work against knot untying in protein unfolding. In topology, the trefoil knot in YbeA is formed by threading α5 through a loop formed by several β-sheets, which also constitute the hydrophobic core of the protein (Figure 1). As revealed in our simulations, separation between domains α1 and α5 is the first step of protein unfolding through opening the hydrophobic core composed of β-sheets. Upon exposure of the hydrophobic core to water, we found denaturation and separation of these β-sheets that caused fluctuations of the knot position and size (Figure 2). In particular, β1-β4 interactions cooperate with α1-α5 interactions to maintain integration of the knotted region with the unknotted region (Figure 1). Upon β1-β4 separation, β4 is less stable and readily denatured and separate from β3 to loosen the loop and allow the knot slide slightly toward the N-terminal. In contrast, the knot end point sliding toward the C-terminal is more difficult due to the rigid turning of α5 by residues Pro128 and Pro130. Besides, β3 and α3 provide one aromatic Trp each to form strong π-π interactions with Trp120 in β5 to stabilize crossing of α5 through the loop. Thus, residues involved in these barriers are strongly correlated in space, and the trefoil knot in YbeA can be occasionally untied if all these barriers are overcome. Otherwise, the protein would stay knotted after denaturation, as observed in previous experiments [19,69].

### 3.5. Site Mutations Manifesting Barriers against Knot Untying

**Figure 6:**
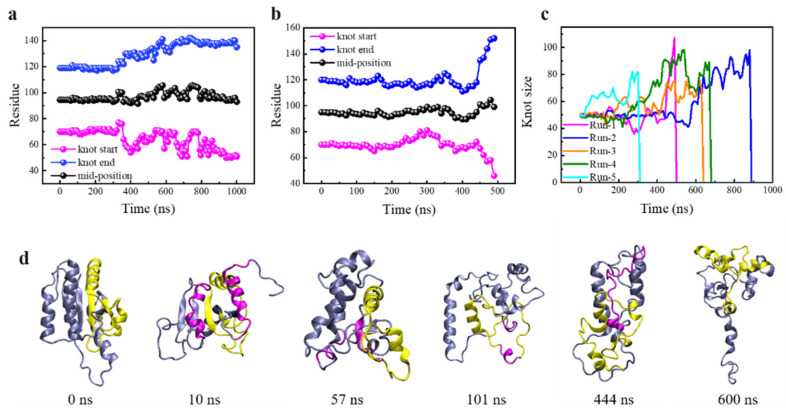
Thermal denaturation of knotted protein YbeA with specific residues mutated. (**a**) Time evolutions of the knot termini and mid-position change in YbeA with two residues Pro128 and Pro130 mutated to Ala. (**b**) The knot position change in YbeA with three residues Pro128, Pro130 and Trp120 mutated to Ala. (**c**) Time evolutions of the knot size in YbeA with three residues mutated in five independent simulations under the same thermal denaturing condition. (**d**) Time sequence of typical snapshots in one simulation depicting the detailed processes of protein unfolding and knot untying. The knotted region is colored in yellow. The major secondary structural change at each step is highlighted by purple coloring.

With four aspects of barriers identified to impede knot untying in protein unfolding, site mutations were then designed to further manifest these barriers. Firstly, residues Pro128 and Pro130, which were found to maintain rigid turning of α5 against its sliding out of the loop, were altered to Ala. After energy minimization and equilibration to remove the improper contacts induced by site mutations, unbiased MD simulation under normal conditions was performed for 560 ns. Our results showed that the site mutations did not change the equilibrium protein structures as confirmed by time evolutions of the secondary structural change, native contact ratio and RMSD (Figure S12). No obvious change in the knot position and size was observed (Figure S13a), suggesting stability of the knot under normal conditions. When the temperature was increased to enhance thermal fluctuations, we found apparent loosening of the knot (Figure 6a), and expected knot untying in a prolonged simulation time. Next, we further mutated Trp120, which was found to form strong π-π interactions with Trp79 and Trp91 to stabilize the loop. Again, no change in protein size and position was observed under normal conditions (Figure S13b). Then, five independent simulations were performed under enhanced thermal denaturing conditions, and surprisingly the knot was untied in all simulation runs (Figure 6b,c and Figure S14). The detailed process was depicted in Figure 6d. Besides the α1-α5 separation opening the hydrophobic core as the first step of protein unfolding, mutations of Pro128 and Pro130 made turning of α5 less rigid and readily deformed to slide out of the loop. Moreover, π-π interactions between Trp120, Trp79 and Trp91 were perturbed by mutation of Trp120, thus making the loop less stable and readily loosened to accomplish the knot untying in finite simulation time.

We chose the protein with three residues (Trp120, Pro128 and Pro130) mutated to Ala and performed steered MD simulations using the same protocol. By comparing time evolutions of the resistance force, we found easier mechanical unfolding of the mutated protein and easier translocation through the tube, as reflected in the lower force peaks (Figure S15a). In particular, we have found tightening of the knot that resisted sliding toward the C-terminal, as ascribed to the rigid turning of α5 by residues Pro128 and Pro130 and the π-π interactions between aromatic residues Trp120, Trp79 and Trp91. With these residues mutated to Ala, as expected, we found sliding of the knot in eight residues toward the C-terminal (Figure S15b). Therefore, mutations of these residues can effectively reduce the barriers to ease the knot untying and sliding under thermal and mechanical denaturing conditions.

## 4. Conclusions

Despite remarkable progress in accurate prediction of most proteins utilizing advanced artificial intelligence models, the dynamic information about folding/unfolding of relatively rare, knotted proteins is lacking. In this study, we have applied the atomistic MD simulation to investigate the thermal and mechanical unfolding of a trefoil knotted protein, YbeA. Our results showed that the knot of YbeA can be occasionally untied through knot loosening under enhanced thermal fluctuations. Through analyzing pathways of protein unfolding in spatial correlations with changes in the knot position and size, four aspects of intramolecular barriers that jointly suppressed knot untying in protein unfolding were revealed. The trefoil knot in YbeA is formed by threading α5 through a loop formed by four β-sheets, which also constituted the hydrophobic core of the protein. Thus, perturbation of α1–α5 interactions was identified as the first step of protein unfolding through opening of the hydrophobic core and exposing it to water. Inside the loop, hydrophobic interactions and hydrogen bonds are formed to stabilize the loop against loosening. In particular, favorable interactions between β3 and β4 can restrict the size of the loop to impede sliding of the knot toward either direction. β1–β4 interactions further enhance the loop stability against separation between the knotted region and the unknotted region. Residues Pro128 and Pro130 at N-terminal of α5 define a rigid turning that restrains α5 from sliding out of the loop. These barriers are spatially inter-connected and cooperatively work to impede the knot from untying in protein unfolding. We designed site mutations to reduce the specific barriers and achieved easier knot untying and sliding in thermal and mechanical protein unfolding. This mechanistic study provides new molecular level insights into protein unfolding decoupled with knot untying, and help reveal the interplay between folding and knotting of proteins similar to YbeA.

## Data Availability

The data presented in this study are available in the Supplementary Materials.

## Supplementary Materials

The following are available online at https://www.mdpi.com/article/10.3390/biom11111688/s1, Figure S1: Structural stability of monomeric YbeA under normal conditions; Figure S2: Time evolutions of the native contact ratio and RMSD of YbeA in five independent simulations under enhanced thermal denaturing conditions; Figure S3: Time evolutions of the number of residues with secondary structures of α helix and β sheet in five independent simulations under enhanced thermal denaturing conditions and one simulation under normal conditions; Figure S4: Time evolutions of the knot size of YbeA in five independent simulations under enhanced thermal denaturing conditions; Figure S5: Thermal denaturation analysis of YbeA in simulation run 1; Figure S6: Thermal denaturation analysis of YbeA in simulation run 2; Figure S7: Thermal denaturation analysis of YbeA in simulation run 3; Figure S8: Thermal denaturation analysis of YbeA in simulation run 4; Figure S9: Distribution of dihedral formed by residues Pro128, Pro130 and Arg133 in equilibrium simulation; Figure S10: Time evolutions of the protein’s secondary structure changes as the N- and C-terminal was pulled through the SWCNT in a constant velocity of 0.0005 nm/ps; Figure S11: Time evolutions of distances between residues Trp91 and Trp120, and residues Trp79 and Trp120 when the N- and C-termini were pulled through the tube; Figure S12: Structural stability of mutated proteins under normal conditions; Figure S13: Sequential movement of two knot termini and mid-position for mutated YbeA mutant with two (Pro128 and Pro130) and three (Pro128, Pro130 and Trp120) residues mutated to Ala under normal conditions; Figure S14: Sequential movement of two knot termini and mid-position for the mutated protein in four independent simulations of thermal denaturation; Figure S15: Effect of mutation on mechanical response of the protein as the N-terminal was pulled through the SWCNT; Video S1: The continuous process of knot untying during protein unfolding under enhanced thermal denaturing conditions; Video S2: The steered protein translocation through SWCNT from the N-terminal; Video S3: The steered protein translocation through SWCNT from the C-terminal.
