# Supplementary material for: Revealing Topological Barriers against Knot Untying in Thermal and Mechanical Protein Unfolding by Molecular Dynamics Simulations
Source: Biomolecules. 2021 Nov 13;11(11):1688. doi: 10.3390/biom11111688 (PMC8615548; doi:10.3390/biom11111688)
Supplement: Supplementary file 1 [file biomolecules-11-01688-s001.zip › biomolecules-1421011-supplementary.pdf]

SUPPORTING MATERIAL

Revealing Topological Barriers Against Knot Untying in Thermal and Mechanical Protein Unfolding by Molecular Dynamics Simulations

Yan Xu,[1,2] Runshan Kang,[2] Luyao Ren,[3] Lin Yang,[3] and Tongtao Yue[3,4,*]

[1]College of Electronic Engineering and Automation, Shandong University of Science and Technology, Qingdao 266590, China

[2]College of Chemical Engineering, China University of Petroleum (East China), Qingdao 266580, China

[3]Institute of Coastal Environmental Pollution Control, Key Laboratory of Marine Environment and Ecology, Ministry of Education, College of Environmental Science and Engineering, Ocean University of China, Qingdao 266100, China

[4]Laboratory for Marine Ecology and Environmental Science, Qingdao National Laboratory for Marine Science and Technology, Qingdao 266237, China

*Correspondence: yuetongtao@ouc.edu.cn

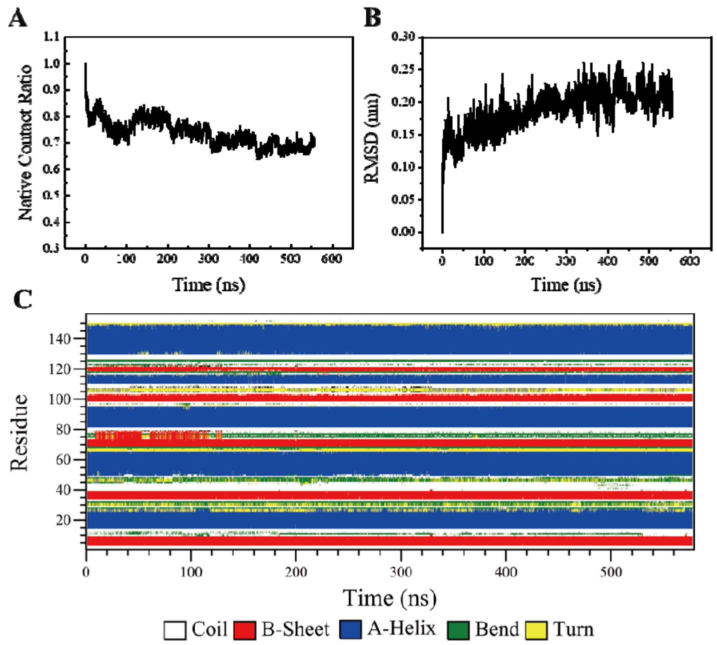

Figure S1. Structural stability of monomeric YbeA under normal conditions. (A) Time evolution of the native contact ratio in YbeA equilibrium simulation. (B) Time evolution of RMSD in YbeA equilibrium simulation. (C) Time evolution of the secondary structural change in YbeA equilibrium simulation.

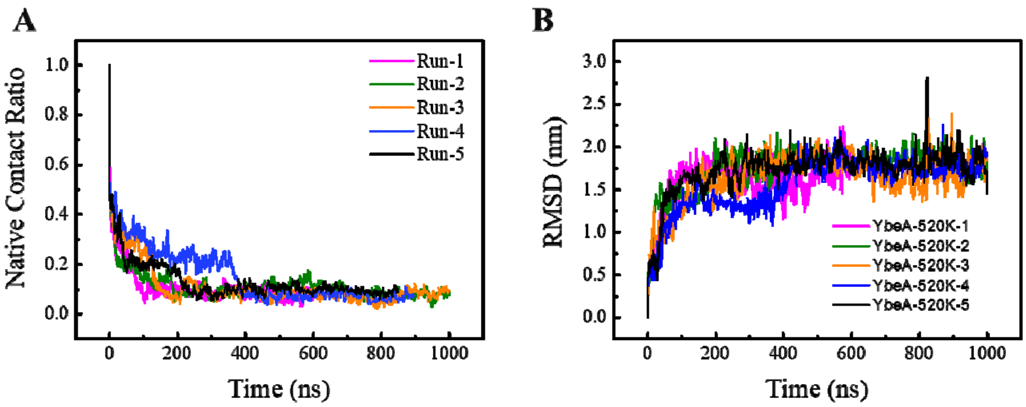

Figure S2. Time evolutions of the native contact ratio (A) and RMSD (B) of YbeA in 5 independent simulations under enhanced thermal denaturing conditions.

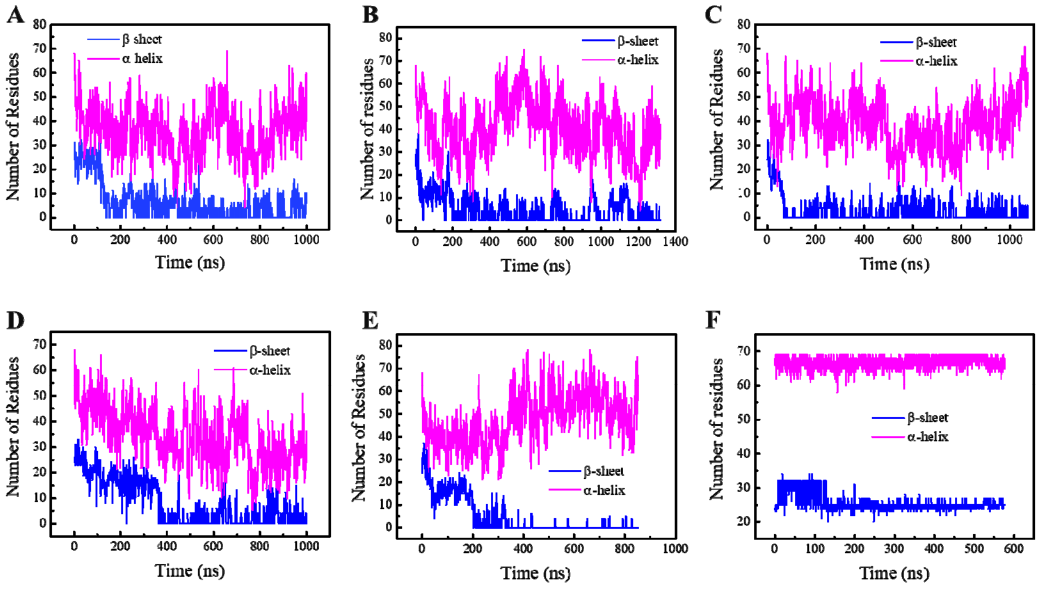

Figure S3. Time evolutions of the number of residues with secondary structures of α helix and β sheet in 5 independent simulations under enhanced thermal denaturing conditions (A–E) and 1 simulation under normal conditions (F).

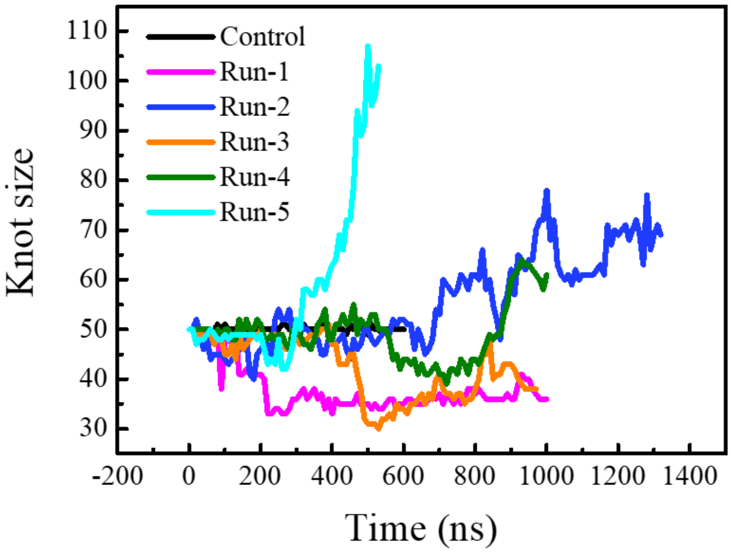

Figure S4. Time evolutions of the knot size of YbeA in 5 independent simulations under enhanced thermal denaturing conditions.

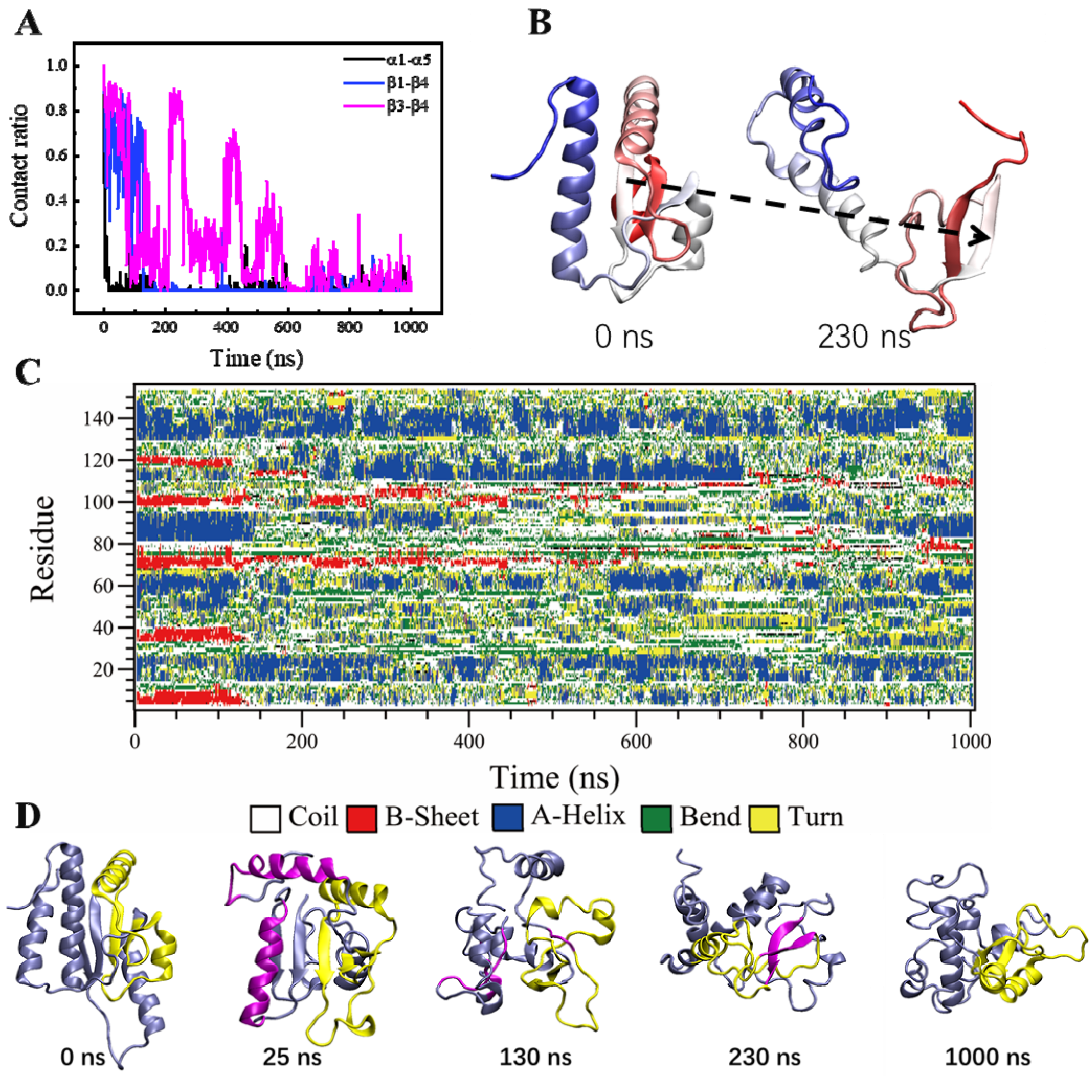

Figure S5. Thermal denaturation analysis of YbeA in simulation run1. (A) Time evolution of the ratio of native contacts between domains α1 and α5, β1 and β4, β3 and β4. (B) Locally enlarged structures at two time points illustrating the contact between β3 and β4 during the simulation. (C) Time evolution of the secondary structural change. (D) Time sequence of typical snapshots depicting the thermal denaturation process. The knotted region is colored in yellow. The major secondary structural change at each step is highlighted by coloring in purple.

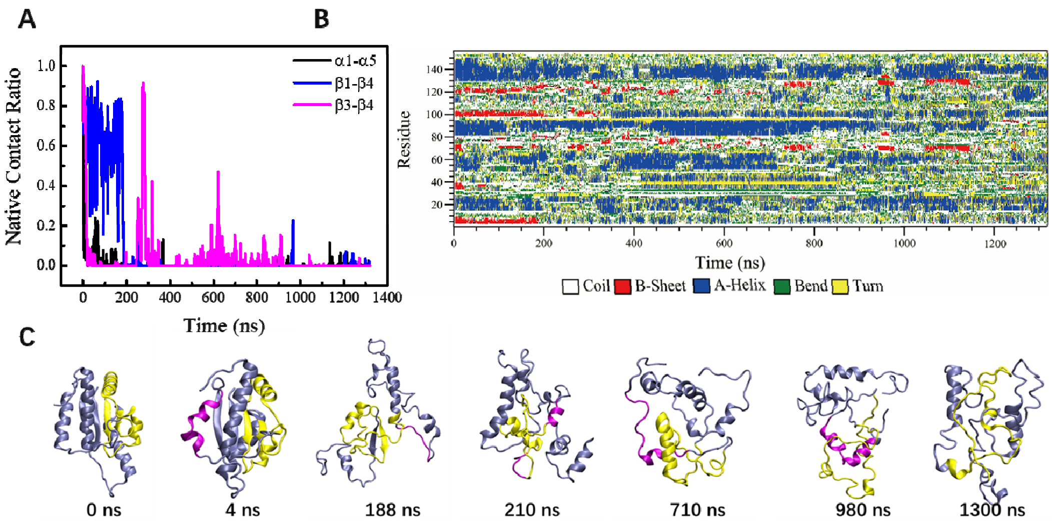

Figure S6. Thermal denaturation analysis of YbeA in simulation run2. (A) Time evolution of the ratio of native contacts between domains α1 and α5, β1 and β4, β3 and β4. (B) Time evolution of the secondary structural change. (C) Time sequence of typical snapshots depicting the thermal denaturation process. The knotted region is colored in yellow. The major secondary structural change at each step is highlighted by coloring in purple.

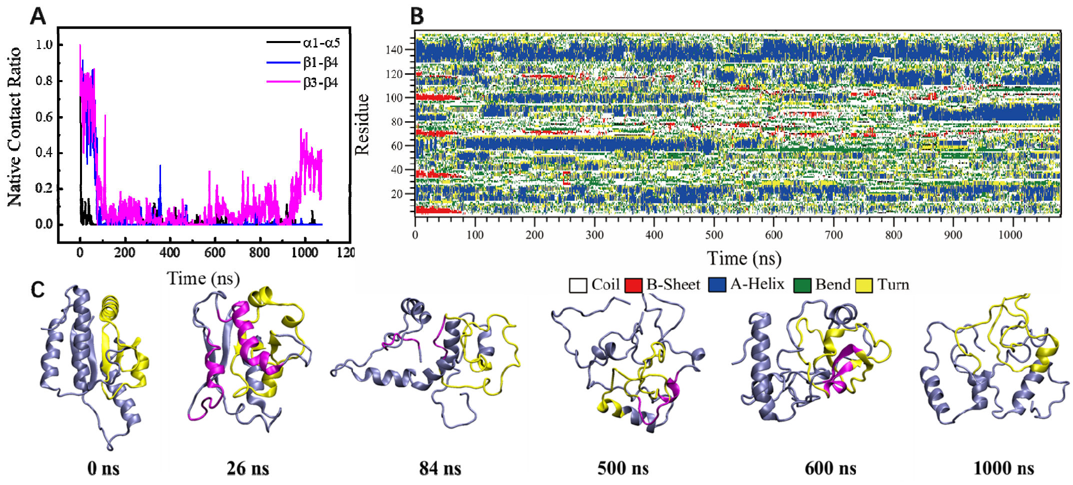

Figure S7. Thermal denaturation analysis of YbeA in simulation run3. (A) Time evolution of the ratio of native contacts between domainsα1 and α5, β1 and β4, β3 and β4. (B) Time evolution of the secondary structural change. (C) Time sequence of typical snapshots depicting the thermal denaturation process. The knotted region is colored in yellow. The major secondary structural change at each step is highlighted by coloring in purple.

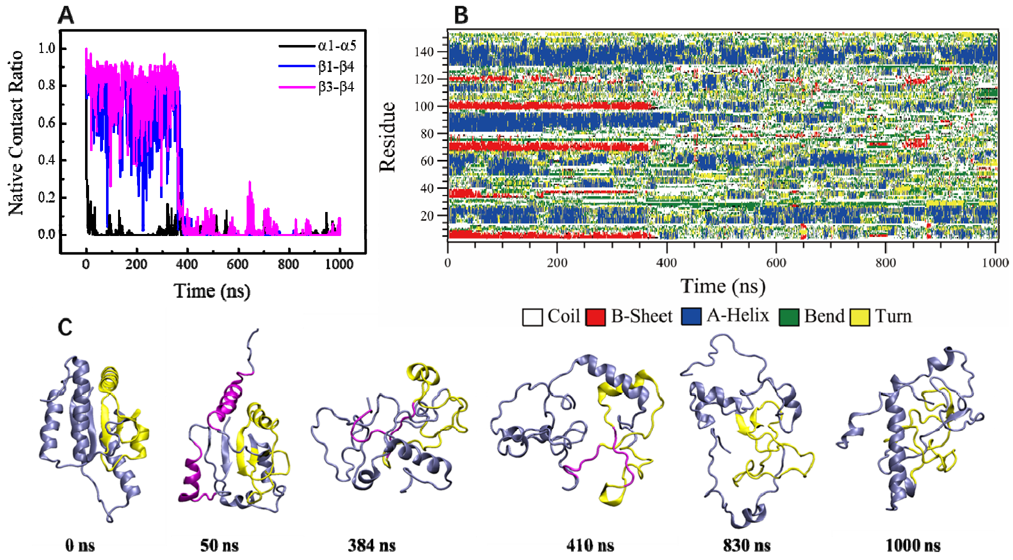

Figure S8. Thermal denaturation analysis of YbeA in simulation run4. (A) Time evolutions of the ratio of native contacts between domains α1 and α5, β1 and β4, β3 and β4. (B) Time evolutions of the secondary structural change. (C) Time sequence of typical snapshots depicting the thermal denaturation process. The knotted region is colored in yellow. The major secondary structural change at each step is highlighted by coloring in purple.

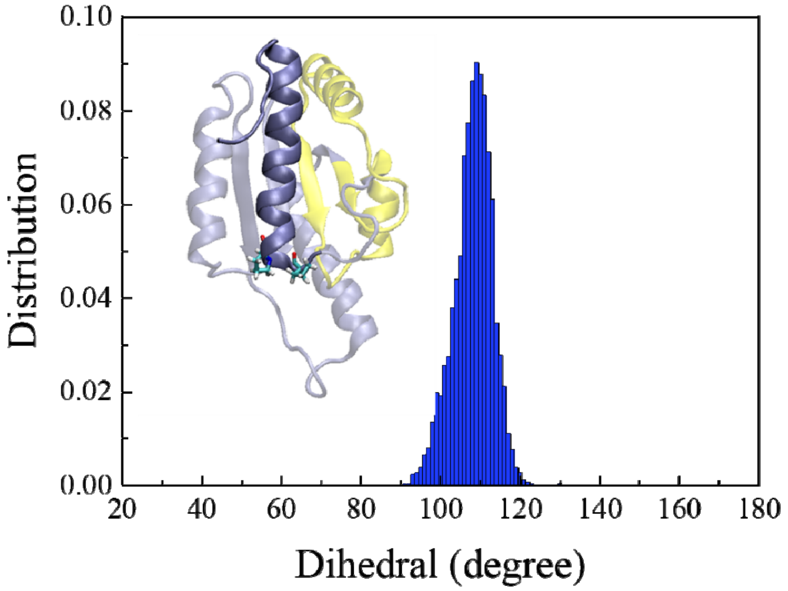

Figure S9. Distribution of dihedral formed by residues Pro128, Pro130 and Arg133 in equilibrium simulation. The inset shows Pro128 and Pro130.

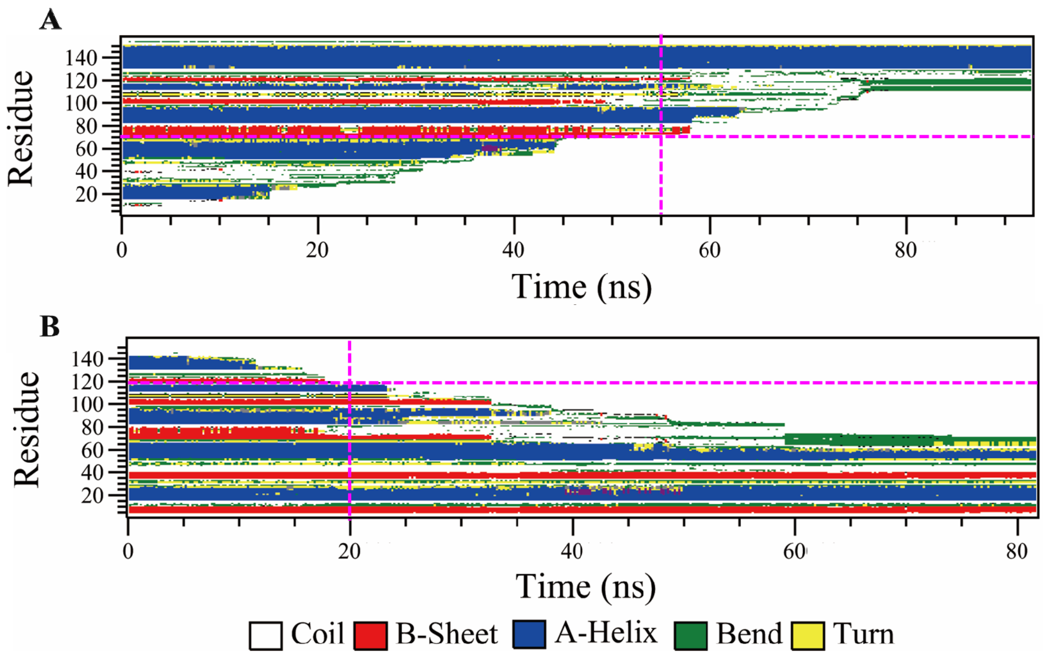

Figure S10. Time evolutions of the protein's secondary structure changes as the N- and C-terminal was pulled through the SWCNT in a constant velocity of 0.0005 nm/ps. (A,B) Two orthogonal dashed lines in each Figure represents the location of one point of the knot and corresponding time point for this point reaching the tube mouth.

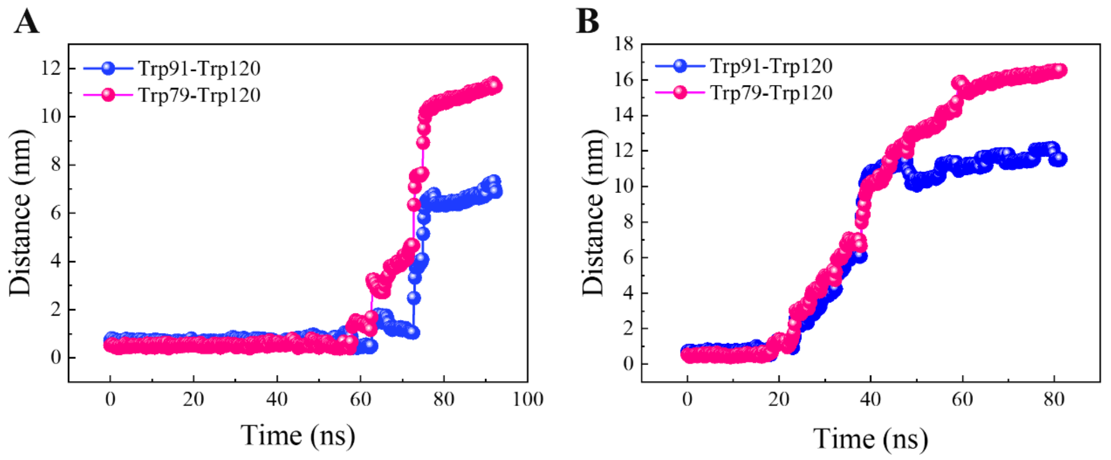

Figure S11. Time evolutions of distances between residues Trp91 and Trp120, and residues Trp79 and Trp120 when the N- (A) and C-termini (B) were pulled through the tube.

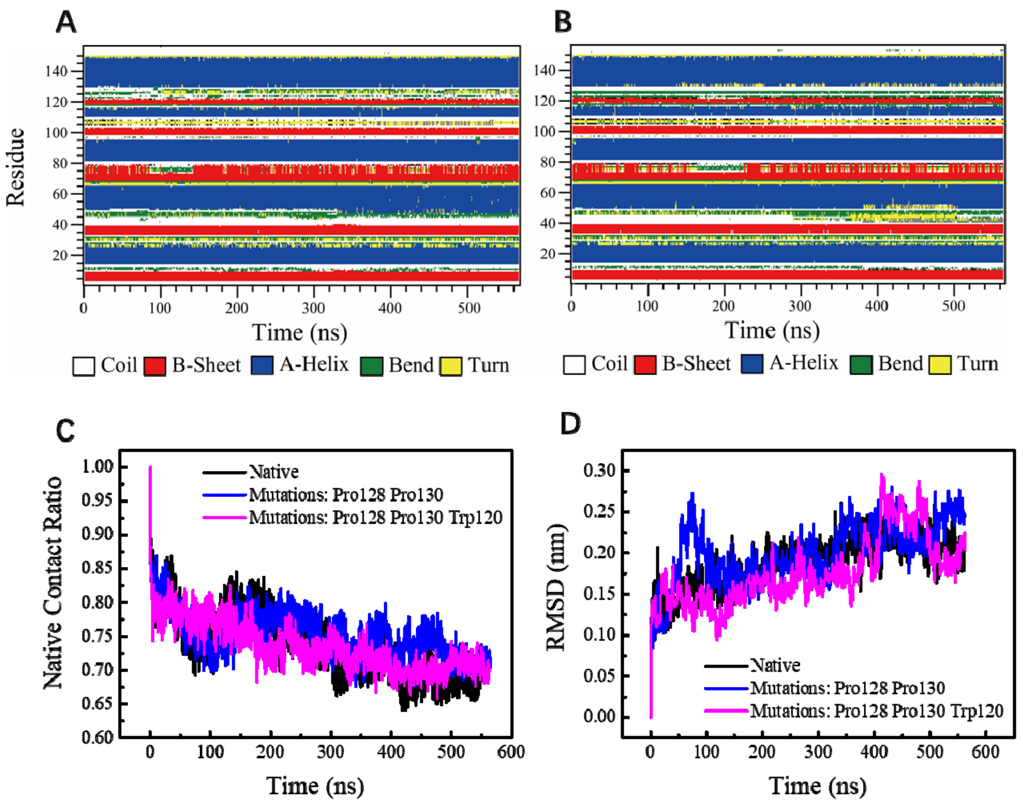

Figure S12. Structural stability of mutated proteins under normal conditions. (A,B) Time evolutions of the secondary structural changes for proteins with two (Pro128 and Pro130, A) and three residues (Pro128, Pro130 and Trp120, B) mutated to Ala. (C) Time evolutions of the native contact ratio for the native and mutated proteins. (D) Time evolutions of RMSD for the native and mutated proteins.

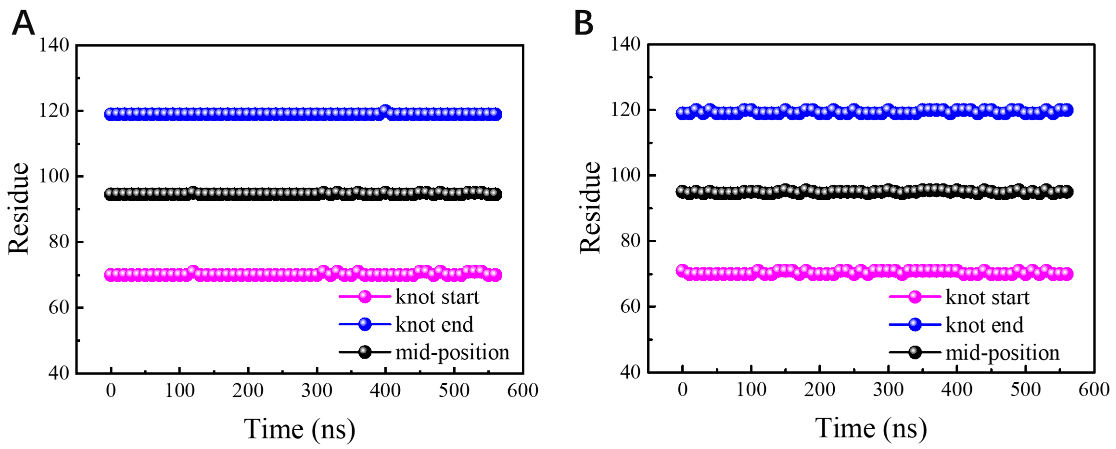

Figure S13. Sequential movement of two knot termini and mid-position for mutated YbeA mutant with 2 (Pro128 and Pro130, A) and 3 (Pro128, Pro130 and Trp120, B) residues mutated to Ala under normal conditions.

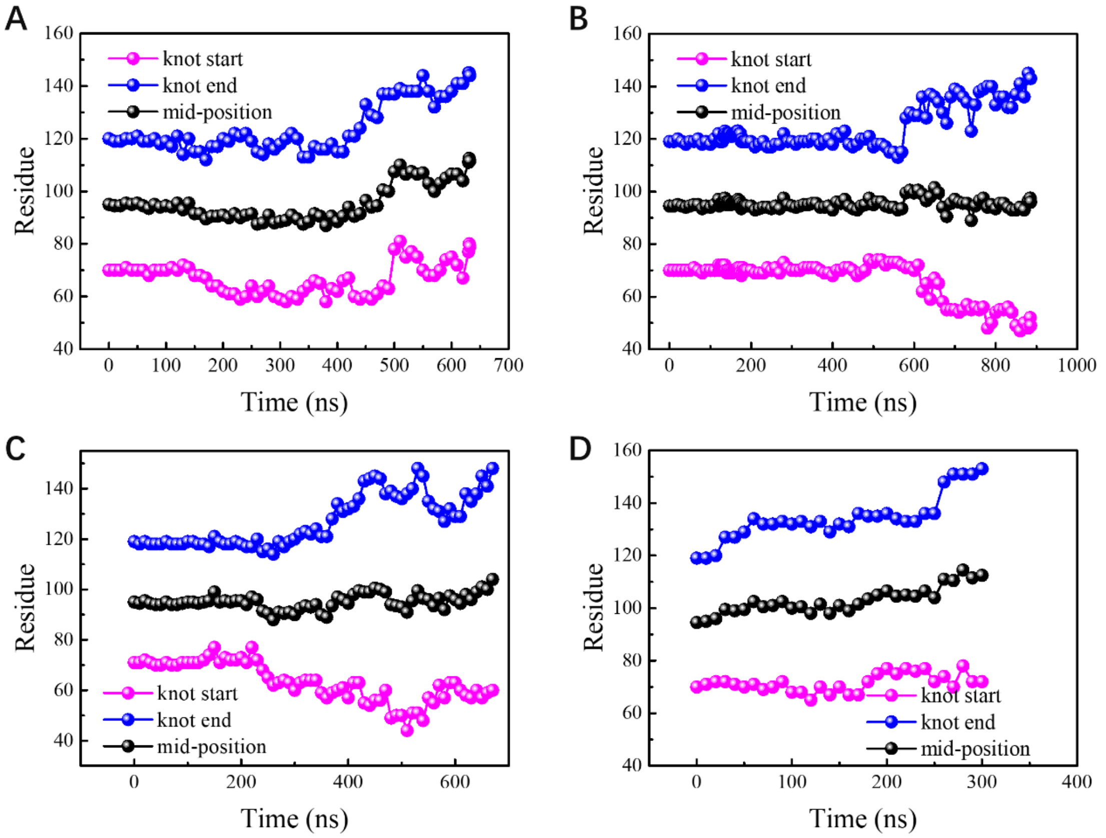

Figure S14. Sequential movement of two knot termini and mid-position for the mutated protein in four independent simulations of thermal denaturation (A–D).

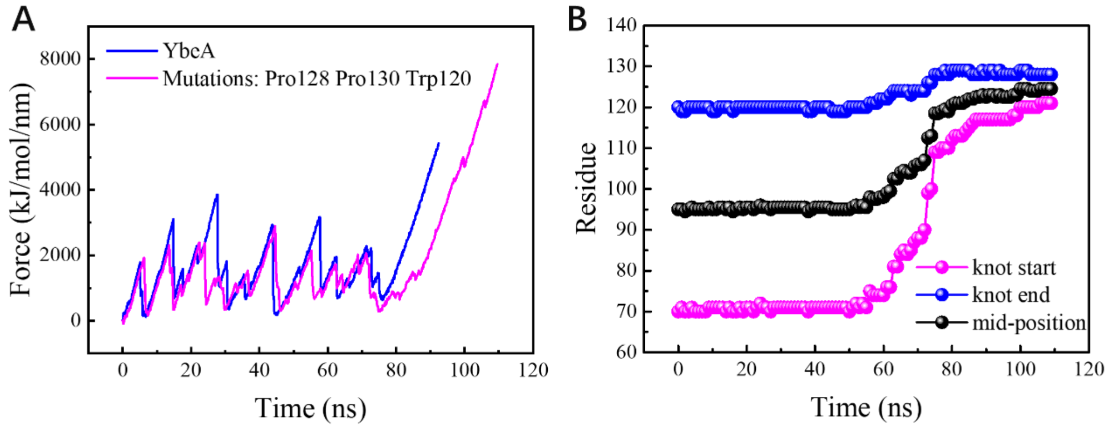

Figure S15. Effect of mutation on mechanical response of the protein as the N-terminal was pulled through the SWCNT. (A) Time evolutions of pulling resistant force as the wild and mutated protein were pulled through the SWCNT from their N-termini. (B) Sequential movement of two knot termini and mid-position for the mutated protein.
